# Puerperal Mania in Bovines1Read before the eleventh annual meeting of the Missouri Veterinary Medical Association, St. Louis, August 18, 1902.

**Published:** 1902-09

**Authors:** E. M. Nighbert

**Affiliations:** Kansas City, Mo.


### PUERPERAL MANIA IN BOVINES.[1]

By E. M. Nighbert, D.V.S.,
KANSAS CITY, MO.

It is not the purpose of this article to project new ideas or discoveries, but rather to bring before you a subject for consideration, that new ideas may be gained.

My attention was first called to this peculiar affection of cows several years ago by Dr. J. D. Nighbert, of Pittsfield, Ill. Since that time I have observed a few cases in my own practice, which will be related later in this article. Puerperal mania of bovines is a nervous disease, maniacal in character, occurring at the time of parturition, immediately after, or from eight to ten hours to six or eight days, or even two weeks, thereafter. The sexual organs of the cow are in action at intervals, and during these intervals of action they diffuse an unusual excitement throughout the nervous system, which is noticed during the catamenial period, occurring at the time of parturition.

*Cause.* Predisposition, as seen in the nervous Jersey and black Yole, is, of course, hereditary, and mental deviations during gestation render an attack of puerperal mania extremely probable. As to the exact pathology of this peculiar disease, it is not very easy to speak positively. From its occurring immediately or soon after parturition, some have attributed it to disease of the uterine system, as new-growths, retention of the lochia, with decomposition, etc. It is contended that in ordinary cases puerperal mania does not arise from inflammation of the brain and its coverings, as in the uterus, which post-mortem examinations on human subjects tend to demonstrate. I am of the opinion that it is neither a congestion nor inflammation of either the brain or uterus, but it is an auto-intoxication the result of changes in the contents of either the alimentary canal, uterus, or of the lacteal secretion. I find upon investigation that the presence in the blood of abnormal constituents is the most important extrinsic cause of nervous disease, and we may consider the subject under the following headings: (1) Poisons produced within the body, by perverted functions of the organs or tissues (auto-intoxication), or by the action of micro-organisms upon the living fluids and tissues of the body; (2) poisons introduced into the body from without. Now, with the constipation that is always

[1] Read before the eleventh annual meeting of the Missouri Veterinary Medical Association, St. Louis, August 18, 1902.

present, and the changes in the milk secretion, and the presence in many cases of retained and decomposed uterine secretions, it tends to bear out the theory given. As I find in looking over veterinary literature very little said of value, I perhaps can make the subject more clear by describing a few cases.

Case I.—Jersey cow, aged three or four years, was turned to pasture two days after calving, in apparently good health. In the afternoon, when driven up, she was noticed to be in distress, as if choked. Upon examination I found: Temperature, 102° F. Heart rapid, with weak pulsations. Respirations increased, but not labored. No tympany on palpation over the rumen, but it was doughy and inactive. Suspension of operation of the bowels well marked. No vaginal discharge, and the vulva well drawn up. Mammary gland flabby, and contained no milk. Eyes prominent, with pupils dilated, which gave the animal a wild or frightened appearance. There was rapid movement of the jaws, with excessive flow of saliva. The animal remained standing, with neck bent upon itself, licking the side continually. She was placed in a stall and one ounce of chloral hydrate administered. Two hours later a brisk purgative of epsom salts was administered; two hours later the patient was found resting naturally, and was left for the night. Next morning was found all right.

Case II.—Jersey cow, seven or eight years old, had given birth to a calf fifteen days previous to attack. The milk was free from colostrum, and it was being fed to a baby, with no bad results, up to two or three days before the attack, when the child was noticed to be restless and fretful, with slight diarrhœa. I was hastily summoned and informed the cow was mad. I found the patient in a frenzied condition, falling upon her knees and licking the ground. She would stand by the fence and lick and bite at the boards. Other symptoms were the same as in Case I. I examined the uterus and found it well contracted, with no growths of any kind, but there was a dark, semigelatinous fluid in the vagina. I washed this out with a creolin solution, ordered a quiet place, and administered chloral hydrate, followed by a physic of salts. Fluid extract of belladonna was administered in small doses at intervals of three hours. After twenty-four hours the patient was discharged. The appetite improved, and the cow did well for two weeks, when the second attack took place. This time I used the chloral and belladonna, with physic, and also washed out the uterus and vagina antiseptically. After two days recovery took place, and the animal remained well. The child suffered no particular inconvenience, and probably the milk from the affected cow was not the cause of the trouble, but it looked as though it might have been.

Case III.—Holstein cow, five or six years old, which had dropped a calf ten days previous to my call; but the history revealed that the cow had not done well since the time of parturition, showing nervous symptoms, and was delirious at times. She recovered from those symptoms, but there was anorexia, and the patient grew anæmic; and owing to an attack of the nervous symptoms I was hastily called a distance of fifteen miles. Upon my arrival I found the peculiar rabiform symptoms, with other complications. There were well-marked uterine changes, the vagina being loaded with decomposed lochia. I pursued the usual course of treatment until the nervousness was over, after which stimulants were administered, followed later by tonics. The cow apparently did well for two or three weeks, when the distressing symptoms returned. My client was a good nurse, and I prescribed by telephone and had him carry out the usual treatment, and the animal recovered, but was three months completing it.

Case IV.—Grade cow, weight about 1200 pounds, well nourished, calved two or three days previously. Was first noticed, early in the morning, to be somewhat uneasy and rather excited. Ate a small quantity of feed, and later was turned out in a small pasture lot, and was not seen until the middle of the afternoon, when it was noticed that she kept her head very low, between the front legs, and apparently tried to put the back of her head or poll on the ground by placing her head far back between the front legs. During intervals of quietude she would hold her head in an almost natural position, but kept up a continual champing of the jaws, with an abundant flow of frothy saliva. Pulse somewhat quickened; temperature elevated two or three degrees. Treatment consisted of a good purge of epsom salts and gamboge, followed by one and a half ounces of chloral; then, every two hours, one drachm of the fluid extract of belladonna, until the nervousness subsided. Next morning the appetite returned, and after the effects of the purge had subsided she was put on her usual amount of feed.

Case V.—Grade shorthorn cow, in good condition, weight about 1000 pounds. Gave birth to her third calf forty-eight hours previously. Owner first noticed her as being somewhat uneasy; wild look out of the eyes, champing of the jaws, and considerable flow of foamy saliva. Every few minutes she would lower her head and lick the inside of her front legs, which were wet with the copious flow of saliva. When an attempt was made to drive her she was inclined to charge the attendant. Pulse and temperature about the same as in Case II. Treatment the same. Recovery in twenty-four hours, though a little dull, perhaps from the effects of medication.

CASE VI.—Grade Jersey cow, weight about nine hundred pounds. Second calf was born twenty-four hours previously. The symptoms were not so well marked as in previous cases. Pupils somewhat dilated; a little excitable. Same peculiar champing of the jaws; not so much inclined to lick the inside of front legs as Case II. Treatment the same as in previous cases. Recovery seemed complete in twenty-four hours.

I wish to say, further, that the puerperal state was normal in all the above cases, delivery was easy, and there was no retention of placenta. The differential diagnosis, I must confess, is puzzling to the beginner, but the characteristic symptoms and the extremely fatal and infectious nature of rabies helps to differentiate that disease, while the sternodecubital position and coma differentiate it from parturient paresis.

---

ON STENOSIS OF THE DUCT IN THE TEAT OF THE COW. Obstruction in the duct of the teat may depend upon spasmodic contraction of the sphincter, to the presence of cicatrices, to the development of papillomata upon the mucous membrane. Those of the mechanical kind are placed in the lower third of the teat. In all cases the author, Eggmann, operates. If the sphincter is too strong or contracts during milking he makes a crucial incision at the summit of the teat (next to the gland); if it is to remedy cicatricial contraction within the teat he introduces a tenotome with a double edge and cuts freely; if there are papillomata he rotates the instrument. The quarter operated upon has then to be carefully treated; into the lumen of the duct he passes a long thread of catgut provided with a sealing-wax button to prevent its passage into the milk sinus. He then slips on the teat a rubber glove-finger of large size. It is first put on the finger and then rolled up on the teat. After about two days the catgut bougie is not replaced after milking, and the glove finger is left off after three to five days. If the incision into the sphincter is too great and the milk flows away, an artificial sphincter is made by putting on a rubber band. Of course, rigid antisepsis is necessary.—*The Veterinarian*, July, 1902.

